# Possible Role of Hydrogen Sulfide in Insulin Secretion and in Development of Insulin Resistance

**DOI:** 10.4103/0975-1483.63156

**Published:** 2010

**Authors:** MA Patel, GB Shah

**Affiliations:** *Department of Pharmacology, C.K Pithawala Institute of Pharmaceutical Science and Research, Near Magdalla port, Surat, India*; 1 *Gujarat, K.B. Institute of Pharmaceutical Education and Research, Sector 23, GH-6, Gandhinagar, Gujarat, India*

**Keywords:** Glucose, insulin, hydrogen sulfide, K^+^ ATP channel

## Abstract

H_2_S has been proposed as physiological important molecule. It is considered as first endogenous gaseous K^+^ channel opener. K^+^ ATP channel activity is mainly responsible for insulin secretion. K^+^ATP channel opening of β cells leads to inhibition of insulin secretion and channels closing leads to secretion. H2S is the gaseous K^+^ ATP channel opener but it does not have channel specificity. So, H_2_S may have some effect on insulin secretion. H_2_S is high in Zuker diabetic fatty rats. That means H_2_S is high in insulin resistance condition. We tried to find out the role of H_2_S in insulin secretion and in development of insulin resistance. From the result of our study, H_2_S have K^+^ ATP channel opening activity on β cells. H_2_S does not have any role in the development of insulin resistance. Decrease in insulin level in Zuker diabetic rat and streptozotocin-induced diabetic rat is due to high H_2_S level.

## INTRODUCTION

In recent years, interest has been directed toward other naturally occurring gases, notably H2S. Now, H_2_S has been proposed as physiological important molecule. Recently, high concentration of H_2_S has been observed in brain of rats, humans, and cows. The H_2_S concentration in rat serum has been found to be 46 μM[1] and in brain tissue 50-160 μM.[2] Significant amount of H2S is produced in various tissues. Cystathionine gamma lyase (CSE), cystathionine β synthase (CBS), and 3-mercaptosulfurtransferase are the enzymes responsible for production of H_2_S.[3] The main subtract for these enzymes is L-cysteine. H_2_S is considered as endogenous vasorelaxant factor and cardiovascular function regulator.[4] Zhao showed that H_2_S is the first gaseous K^+^ATP channel opener.[1] Intravenous injection of H_2_S provoked a transient but significant decrease in mean arterial blood pressure. H_2_S-induced decrease in blood pressure was antagonized by glibenclamide and mimicked by pinacidil.[1] Glibenclamide and pinacidil are specific K_ATP_ channel blocker and opener, respectively. Thus, these *in vivo* results indicated that the hypotensive effect of H_2_S was likely provoked by the relaxation of resistance blood vessels through the opening of K_ATP_ channels. Its mechanism of K^+^_ATP_ channel opening is still unknown, but it does not affect ATP concentration. K^+^_ATP_ channel activity is mainly responsible for insulin secretion. K^+^_ATP_ channel opening of β cells leads to inhibition of insulin secretion and channels closing leads to secretion. H_2_S is the gaseous K^+^ ATP channel opener but it does not have channel specificity. So, H_2_S has some effect on insulin secretion. H_2_S concentration in Zuker diabetic rats was found to be high.[5] That means H_2_S is high in insulin resistance condition. According to Muhammed, experimental diabetes in rats induced by streptozotocin leads to increase expression of the H_2_S producing enzymes.[6] H_2_S may have an inhibitory effect of insulin secretion due to nonspecific K^+^ ATP channel opening activity. On the other side, H_2_S level is high in hyperinsulinemia. So, this study was carried out to investigate the role of H_2_S in insulin secretion and development of insulin resistance.

## MATERIALS AND METHODS

### Animals

Adult albino rats of either sex (Wistar strain) weighing between 200 and 250 g were used for the study. The animals were fed *ad libitum* with standard pellet diet and had free access to water. All experiments and protocols described in present report were approved by the Institutional Animal Ethics Committee (IAEC).

### Experimental protocol

Effect of H_2_S on the K^+^_ATP_ channel of insulin secreting cell was studied by dividing animals into two groups. Group 1 received NaHS 2 mg/kg i.p. (high rate of mortality on higher dose), and Group 2 received only saline. In the second experiment, animals were divided into two groups. Group 1 received saline only and group 2 received glibenclamide 10 mg/kg, p.o[7] then after NaHS 2 mg/kg i.p. Blood was collected from tail vein at 0, 30, 60, 120 min after administration of drugs up to 5 h and blood glucose and insulin were measured. The blood glucose was measured by the GOD/POD[8] method and insulin was measured by RIA.[9]

### Protocol to study the role of H_2_S in insulin resistance development

The wistar albino rats are divided into two groups (*n*=6). One group received NaHS 2 mg/kg i.p twice a day for 30 days. Another group received only saline. The development of insulin resistance was studied by performing oral glucose tolerance test (OGTT). Also OGTT was performed after giving insulin by i.m. to measure sensitivity toward exogenous insulin. Insulin and glucose level were also measured before and after 30 days treatment.

### Oral Glucose Tolerance Test (OGTT)

After 30 days, 5 g/kg of glucose was administered to fasted animals. Blood samples were collected from tail vein under light anesthesia for 0 min and 15, 30, 60, 90 and 120 min after oral glucose administration. The blood samples were allowed to clot and serum was separated by centrifugation. The samples were measured for glucose level by the GOD/POD method.[10]

### Oral Glucose Tolerance Test (OGTT) with Insulin

Wistar rats were divided into two groups of six animals. One is treated with NaHS 2 mg/kg for 30 days other is treated with saline. After 30 days animals were orally administered with 5 g/kg of glucose and insulin 1 U/kg i.m. Blood samples were collected from tail vein under light anesthesia before 0 min and 15, 30, 60, 90 and 120 min after oral glucose administration. The samples were allowed to clot and serum was separated by centrifugation. The samples were measured for glucose level by the GOD/POD method.[10]

### Measurement of serum H_2_S level in streptozotocin-induced diabetic rat

Type II diabetes was induced in wistar rats by streptozotocine.[11] After induction of diabetes, H_2_S level in serum was measured. Seventy-five microliters of plasma were mixed with 250 μl of 1% w/v zinc acetate and 425 μl distilled water. Then 20 mM *N*-dimethyl-*p*-phenylenediamine sulfate in 7.2 mM HCl (133 μl) and 30 mM FeCl_3_ in 1.2 mM HCl (133 μl) were also added to the test tube for 10-min incubation at room temperature. The protein in the plasma was removed by adding 250 μl of 10% tricholoacetic acid to the reaction mixture and pelleted by centrifugation at 14 000 *g* (5 min). The absorbance of the resulting solution at 670 nm was measured with a spectrophotometer (TECAN Systems) in a 96-well plate. All samples were assayed in duplicate, and concentration in the solution was calculated against a calibration curve of NaHS (3.125–250 μM). Results show plasma H_2_S concentration in micromolar[12]

### Statistical analysis

All the values were expressed as mean ± SEM. The statistical analysis was performed using Student’s unpaired *t*-test and by AUC comparison. Value of *P* less than 5% (*P*< 0.05) was considered statistically significant.

## RESULTS

### Effect of H_2_S on insulin and glucose level

After administration of H_2_S (2 mg/kg), glucose insulin level were measured. Administration of H_2_S results in an increase in glucose level and a decrease in insulin level which became normal after 5 h [Table 1]. After administration of glibenclamide 10 mg/kg, p.o H_2_S was given. Insulin and glucose level in animal treated with glibenclamide and H_2_S was not significantly changed compared to saline-treated animals. That means effect of H_2_S was inhibited by glibenclamide [Table 2].

**Table 1:** Effect of H2S on glucose and insulin level

Time (min)	Glucose (mg %)		Insullin(μU/ml)	
	Saline treated	H2S treated	Saline treated	H2S treated
0	79.9±2.7	80.1±6.1	40 ±1.9	41.3 ±1.73
30	85.9±3.6	112.3±7.1*	40.7 ±1.7	34.7±1.5*
60	89.1±5.2	142.7±8.3*	43.7±0.9	30.5 ±1.01*
120	89.6±4.1	174.7±7.3*	43.2±2.4	32.7±2.9*
180	88.7±4	164.2±8.5*	39.8 ±2	33.8 ±2.29*
240	84.4±6.3	132.8±7.9*	41.3±1.2	35 ±1.75*
300	83.8±5.1	116.6±6.9*	41 ±1.5	36.3±1.3*

Each value is mean±SEM

* *P*<0.05

**Table 2:** Effect of glibenclamide on H2S-induced changes on glucose and insulin level

Time (min)	Glucose (mg %)		Insullin(μU/ml)	
	Saline treated group	H2S + glibenclamide-treated group	Saline treated group	H2S + glibenclamide-treated group
0	81.6±2.6	79.5±2.7	38 ±1.9	40 ±0.9
30	83.5±2.9	84.7±3.6	41.6 ±0.7	42.3 ±1.2
60	86.3±4.1	86.6±5.2	43±1.2	41.9±1.3
120	84.2±3.5	85.4±4.1	42.2±1.4	44.1±1.6
180	88.7±3.2	85.7±4	40 ±2	42 ± 1.5
240	85.6±4	83.2±6.3	40.7±0.8	42.7±1.2
300	87.5±3.9	83.8±5.1	40 ±1.5	42±0.8

Each value is mean±SEM, *P<0.05

### Effect of H_2_S on insulin resistance

**Figure 1:**
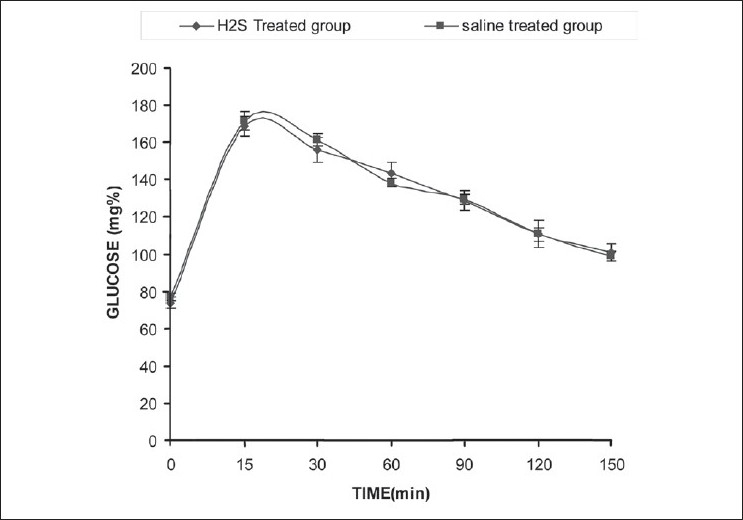
Effect of chronic administration of H2S on oral glucose tolerance. Each value is mean±SEM, *P<0.05

After chronic treatment with H_2_S, oral glucose tolerance test was performed to check the development of insulin resistance. Glucose level was measured after administration of glucose 5 g/kg p.o. There was no significant difference in glucose level compared to normal animals [Figure 1].

**Figure 2:**
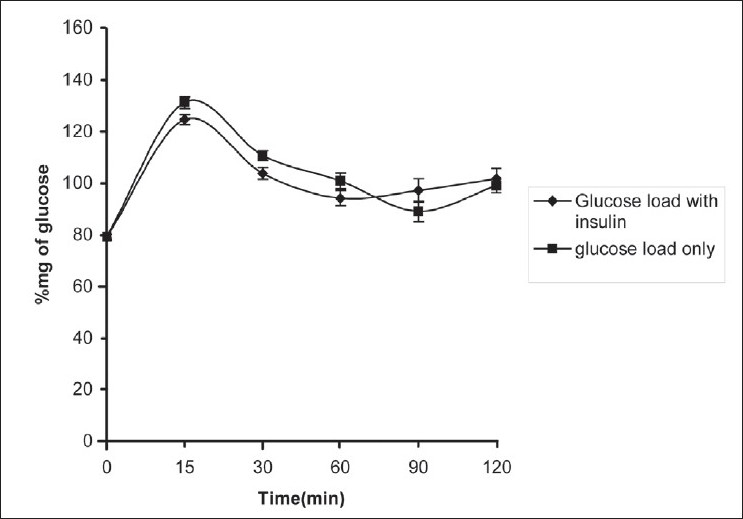
Effect of chronic administration of H2S on oral glucose tolerance. Each value is mean±SEM, *P<0.05

After chronic treatment with H_2_S, oral glucose tolerance test was performed along with administration of insulin 1 U/kg i.m to check the development of insulin sensitivity. Glucose level was measured after administration of glucose 5 g/kg p.o. There was no significant difference in glucose level compared to normal animals [Figure 2]. Also there was no significant difference in insulin and glucose level before and after chronic administration of H_2_S [Table 3].

**Table 3:** insulin and glucose level before and after 30 days treatment of H2S

	Glucose level (mg %)	Insullin(μU/ml)
Before 30 days treatment of H2S	82.4±1.83	42.12±0.54
After 30 days treatment of H2S	82.8±1.12	41.7±0.36

Each value is mean±SEM, *P<0.05

## DISCUSSION

The islets of Langerhans contain four main cell types, out of them β cells secrete insulin. The main factor controlling the synthesis and secretion of insulin is the blood glucose concentration. ATP-sensitive K^+^-channel determines the resting membrane potential in β cells via a membrane transporter called Glut-2 and its subsequent metabolism via glycosidase and glycolysis increases intracellular ATP. This blocks K^+^_ATP_ causing membrane depolarization and opening of voltage dependant calcium channels, leading to Ca^+^ signal induces insulin secretion. The insulin secretion is depending upon K^+^_ATP_ channel opening and closing. H_2_S is K^+^_ATP_ channel opener and it has no effect of ATP concentration, may be acting by direct interaction with protein.[13] So, it may have effect on pancreatic K^+^_ATP_ channel. To study the effect of H_2_S, NaHS was used as a source to release H_2_S *in vivo*. The effect of H_2_S on insulin and glucose level were studied by giving NaHS 2 mg/kg i.p and the blood samples were collected before and after NaHS administration up to 5 h. The glucose and insulin levels were determined in serum. The glucose level was significantly higher and insulin level was significantly lower in animals treated with the NaHS compared to that in the control group. According to our study, H_2_S increases glucose level and decreases insulin level and this effect of H_2_S was inhibited by glibenclamide. That confirms ATP-dependant K channel opening activity of H_2_S in β cells. According to Jia, H_2_ S concentration was reported to be higher in Zuker diabetic rats as compared to that in and Zuker lean rats. Expressions of both the enzymes responsible for H_2_S production, CSE and CBS, were also high in streptozotocin-induced diabetic rats.[6] The overexpressing of these enzymes was also reported in patients having type II diabetes.[6] Probably H_2_S should be having some role in etiology of insulin resistance. So, direct study was conducted by administering H_2_S to the animals chronically and observing changes in insulin sensitivity. After 30 days treatment with H_2_S, neither insulin nor glucose level got altered. Glucose utilization was also not changed. Thus, chronic treatment of H_2_S for 1 month has no significant effects on insulin sensitivity. H_2_S level was also high in streptozotocin-induced diabetic rats compared to saline-treated rats. High level of H_2_S is because of overexpression of H_2_S forming enzymes.[6] So, H_2_S is not responsible for development of diabetes, but high H_2_S level in diabetes is due to overexpression of H_2_S producing enzyme. This over expression of enzymes is may be due to metabolic disturbance in diabetes. A characteristic of streptozotocin-induced diabetes is strong reduction of the pancreatic insulin stores, decreased basal insulin levels, and lack of plasma insulin response to glucose *in vivo*.[14] This decrease in insulin level and lack of plasma insulin response to glucose may be due to high H_2_S level in these diabetic animals. Also in Zuker diabetic fatty rats insulin level is low, it may be due to high level of H_2_S. During OGTT, the increase in serum insulin level of Zuker diabetic fatty (ZDF) rats was delayed with much lower amplitude as compared with that of ZL rats. Treatment with H_2_S producing enzyme inhibitor, DL-propargylglycine (PPG), elevates the basal level of serum insulin of ZDF rats. Both the rate of increase and the peak amplitude of serum insulin level of PPG-treated ZDF rats were significantly increased in comparison with that of untreated ZDF rats.[15]

## CONCLUSION

From the result of our study, it can be concluded that H_2_S is pancreation K+_ATP_ channel opener and it inhibit insulin secretion. High H_2_S level in streptozotocin-induced diabetic rat and ZDF rat is responsible for the low level of insulin and it does not have any role in development of insulin resistance. So, H_2_S can be a good target to treat diabetes but further detail investigation of role of H_2_S in insulin resistance is require to study.
